# Effect of Mesenchymal Stem Cells and Platelet-Rich Plasma on the Bone Healing of Ovariectomized Rats

**DOI:** 10.1155/2016/9458396

**Published:** 2016-11-23

**Authors:** Bo Wei, Chengshuo Huang, Mingyan Zhao, Peng Li, Xiang Gao, Junchao Kong, Yanru Niu, Rui Huang, Juanhua Quan, Jinsong Wei, Jiaqi Chu

**Affiliations:** ^1^Department of Spinal Surgery, Affiliated Hospital of Guangdong Medical University, Zhanjiang 524001, China; ^2^Stem Cell Research and Cellular Therapy Center, Affiliated Hospital of Guangdong Medical University, Zhanjiang 524001, China; ^3^Department of Gastroenterology, Affiliated Hospital of Guangdong Medical University, Zhanjiang 524001, China

## Abstract

We evaluated the efficacy of platelet-rich plasma (PRP) in combination with allogeneic bone marrow mesenchymal stem cells (BMSCs) for the treatment of osteoporotic bone defects in an ovariectomized rat model. By day 42 after injury,* in vivo* microcomputed tomography (micro-CT) imaging revealed that bone defects of control rats and ovariectomized rats treated with PRP and BMSCs were completely repaired, whereas those of ovariectomized rats treated with PRP or BMSCs alone exhibited slower healing. Histological data were consistent with these results. We also assessed changes to bone trabeculae in the proximal tibial growth plate. In ovariectomized rats treated with PRP or with a combination of PRP and BMSCs, the trabecular connectivity densities (Conn.D), bone volume ratios (BV/TV), and numbers (Tb.N) in the defect areas increased significantly from day 7 to day 42. These results indicate that PRP treatment enhances bone microarchitecture in osteoporosis. Moreover, expression levels of osteogenesis-specific marker genes including RUNX2, OSX, and OPN were significantly upregulated in rats treated with PRP and BMSCs compared to those of other groups. Thus, we conclude that treatment with PRP combined with BMSCs significantly promotes healing of osteoporotic bone defects. This study provides an alternative strategy for the treatment of osteoporotic bone loss.

## 1. Introduction

The treatment of bone defects, especially in the case of osteoporosis, remains controversial. The “gold standard” for the treatment of bone defects is autogenous bone grafting, but this method is constrained by the problem of limited bone mass and the additional trauma caused by the surgical site [1]. Allogeneic bone transplantation can solve problems related to the source of the bone graft. However, while transplanted bone can provide a scaffold for osteoconduction and residual bone morphogenetic protein can promote bone healing, strong immune responses limit the clinical application of allograft bone transplantation [2]. Previous studies have demonstrated that bone healing in postmenopausal osteoporotic women and estrogen depletion-induced osteoporotic animals is remarkably delayed or impaired [3]. The number of patients with osteoporotic fractures or bone defects is gradually increasing along with the age of the population. It is therefore necessary to find a more effective treatment for osteoporosis and the healing of bone defects.

It has been suggested that age-related osteoporosis is associated with an increased propensity toward adipocyte differentiation accompanied by a reduction in the osteogenesis of bone marrow mesenchymal stem cells (BMSCs) [4]. An alternative strategy for the treatment of osteoporosis could therefore involve enhancing the proliferation and osteogenic differentiation potential of BMSCs [5, 6]. Activated platelet-rich plasma (PRP) releases several growth factors associated with wound healing and bone regeneration, including transforming growth factor-*β*1 (TGF-*β*1), insulin-like growth factor, platelet-derived growth factor, basic fibroblast growth factor, and vascular endothelial growth factor [7]. Recent studies have established that PRP treatment could improve overall bone quality in osteoporotic mice by promoting osteogenesis while suppressing adipogenesis in bone marrow [8]. Moreover, PRP can stimulate the differentiation of embryonic fibroblasts into osteoblast-like cells; the transplantation of these PRP-treated cells significantly improved bone architecture in osteoporotic mice [9]. It has also been demonstrated that PRP treatment combined with BMSCs may enhance the formation of new bone [10, 11]. However, whether this treatment is effective in the case of osteoporosis remains unknown.

Many molecules play important roles in the regulation of bone formation. The phosphorylation of runt-related transcription factor 2 (RUNX2) and the induction of osterix (OSX) gene expression and alkaline phosphatase activity are associated with bone formation [12]. Osteopontin (OPN) is a prominent constituent of the bone matrix. These molecules are often used as markers in the evaluation of osteogenic differentiation* in vitro* and* in vivo* [13]. Peroxisome proliferator-activated receptor gamma 2 (PPAR*γ*2) has been described as an adipocyte-specific marker [14]. Thus, these markers can be used in monitoring the balance between osteogenesis and adipogenesis of BMSCs* in vivo*.

Collectively, these observations led us to hypothesize that treatment with BMSCs combined with PRP may improve bone regeneration in osteoporotic bone injuries. To test this hypothesis, we used BMSCs and PRP derived from Sprague Dawley (SD) rats to treat bone defects of ovariectomized rats. Callus formation and mineralization were constantly monitored using microcomputed tomography (micro-CT) analysis. Quantitative real-time PCR was employed for determining the expression levels of bone healing related genes. Decalcified histology was used to describe callus histopathology features at the tissue level.

## 2. Materials and Methods

All procedures involving animals were approved by the Animal Care and Experiment Committee of Guangdong Medical University and complied with the Guide for the Care and Use of Laboratory Animals by the National Institutes of Health.

### 2.1. Preparation and Treatment of Thrombin-Activated PRP

Rat PRP releasate was prepared as previously described [11] with some modifications. Briefly, five female healthy SD rats (210–290 g) were anesthetized with chloral hydrate (400 mg/kg i.p.), and one pool of blood was collected from the heart in tubes that were rinsed with heparin. Blood was centrifuged twice: first at 215 ×g for 10 min at 20°C to remove red blood cells and then at 863 ×g for 10 min at 20°C to obtain PRP. The PRP was then activated with one unit per mL rat thrombin (Sigma-Aldrich, Saint Louis, MO, USA). After activation, the PRP releasate was separated from the cellular debris by centrifugation at 3000 ×g for 20 min at 4°C, followed by filtration through a 0.2 *μ*m filter. The PRP releasate was aliquoted and stored at −80°C until usage. To ensure the consistency in PRP utilization, the TGF-*β*1 concentration within the PRP releasate was determined by a rat TGF-*β*1 ELISA kit according to the manufacturer's protocol (BD Biosciences, San Diego, USA) and used as an indicator for quantifying PRP as described elsewhere [9, 15]. The concentration of TGF-*β*1 within the PRP releasate was 102.12 ng/mL as assessed by ELISA assays. A final concentration of 750 pg/mL of TGF-*β*1 in PRP-treated cells or rats (i.e., the PRP-containing medium was calibrated on the basis of a TGF-*β*1 concentration of 750 pg/mL) was employed throughout the study, as it has been shown that rat BMSCs proliferate under such a condition [16].

### 2.2. Isolation and Culture of Rat BMSCs

Rat BMSCs were isolated and expanded using previously reported methods with minor modifications [17]. In brief, bone marrow was collected from the femur and tibia of 30-day-old SD rats. Bone marrow cells were flushed and centrifuged in a 1.073-g/mL Percoll (Pharmacia, Uppsala, Sweden) density gradient. Enriched cells were collected from the interface and transferred into culture flasks with Dulbecco's modified Eagle's medium-low glucose (DMEM, Gibco, Grand Island, NY, USA) supplemented with 10% fetal bovine serum (Gibco) at 37°C and 5% CO_2_. After seeding for 72 h, nonadherent cells were discarded, and adherent cells were washed twice with phosphate-buffered saline (PBS). Fresh complete medium was added and replaced every 3 d. To identify their multilineage differentiation potentials, cells were induced to differentiate into adipocytes and osteoblasts, and their differentiation capacities were measured by staining with oil red O or alizarin red using Oricell differentiation kits (Cyagen Biosciences, Santa Clara, CA, USA) following the manufacturer's instructions. For phenotypic characterization, harvested cells were incubated with phycoerythrin- (PE-) labeled antibodies (BD Biosciences, San Jose, CA, USA) against CD29, CD31, CD34, CD44, CD45, or CD90 and assayed by FACSCanto II flow cytometer (BD Biosciences). Rat BMSCs from passages 2–6 were used for subsequent experiments.

### 2.3. Proliferation and Colony-Forming Assays

The cell proliferation was assessed by using the MTS cell viability assay kits (Promega, Madison, WI, USA) according to the supplier's instructions. Briefly, 5000 cells were placed per well of a 96-well plate and incubated in complete culture media supplemented with PRP for 48 h. The cells grown in complete culture media without any treatment were used as controls. The colony-forming assay was carried out as previously described in [16].

### 2.4. Animals and Surgical Procedures

**Figure 1:**
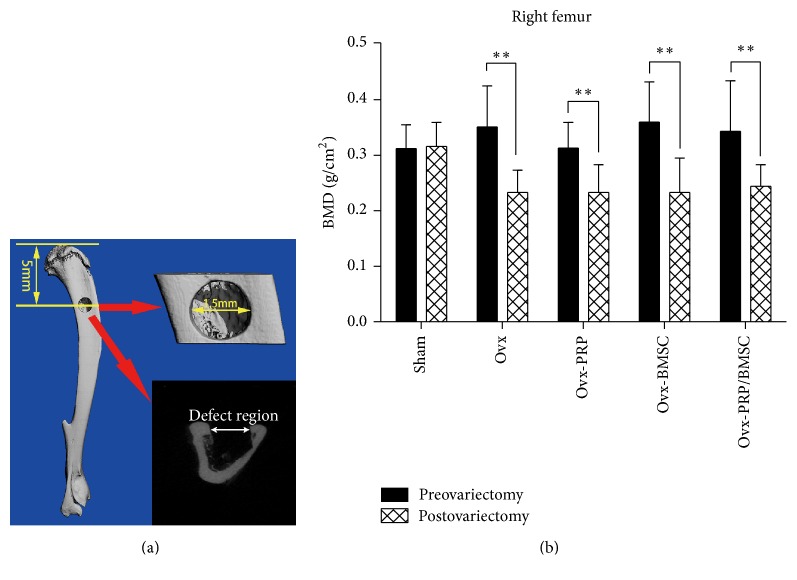
Ovariectomized bone defect animal model. (a) Schematic representation of the drill-hole defect area using micro-CT analysis. The drill-hole is located in the lateral plane of the tibial plateau. The diameter of the tibial drill-hole was 1.5 mm, which penetrated one side of the bone cortex. (b) Bone mass densities of rats before and after ovariectomy (^*∗∗*^ *P* < 0.01, postovariectomy versus preovariectomy).

Female rats were purchased from the Guangdong Medical Laboratory Animal Center of China with an initial weight of 200 ± 20 g. All animals were randomly assigned to five treatment groups (20 rats in each group): the sham operation group, in which rats received PBS; the Ovx group, in which rats were ovariectomized and then received PBS; the Ovx-BMSCs group, in which rats were ovariectomized and then treated with BMSCs; the Ovx-PRP group, in which rats were ovariectomized and then treated with PRP; and the Ovx-PRP/BMSC group, in which rats were ovariectomized and then received PRP and BMSCs. A bone mineral density (BMD) test was performed using a Bruker Xtreme* In Vivo* Imaging System (Bruker, Billerica, MA, USA) just before ovariectomy. The ovariectomy and sham operation protocols were carried out as previously described [4]. Rats were allowed to feed conventionally for three months after ovariectomy. BMD was again assessed to confirm the osteoporotic animal model. A 1.5 mm diameter tibial defect was made in all SD rats approximately 5 mm from the proximal tibial growth plate by hollow drill (Figure 1(a)). The same leg was used for each group. Holes were rinsed by injection of saline to remove bone fragments from the cavity. Subsequently, the defect areas were filled with PBS, PRP (20 *μ*L), and/or BMSCs (1 × 10^6^ cells), according to experimental group. Five postoperative rats from each group were sacrificed under general anesthesia with intraperitoneal injection of 1% pentobarbital sodium (0.1 mL/100 g) on days 7, 14, 28, and 42, and tibias were harvested. Samples were assessed using micro-CT, histological staining, and real-time RT-PCR (see below) at each time point.

### 2.5. *In Vivo* Micro-CT Imaging and Analysis

Using* in vivo* micro-CT imaging (vivaCT 40, Scanco Medical, Switzerland), changes in trabecular bone morphology and BMD in the defect area and a control area were tracked over time in animals over the course of six weeks. Micro-CT analysis was performed with a source voltage of 70 keV, current of 114 *μ*A, and 10.5 *μ*m isotropic resolution. Parameters including trabecular connectivity density (Conn.D), trabecular bone volume ratio (BV/TV), trabecular number (Tb.N), and trabecular space (Tb.Sp) were calculated from the resulting binary images using the Micro-CT Evaluation program (version 6.1-2).

### 2.6. Quantitative Real-Time PCR

Callus samples were isolated from the defect sites of experimental rats. The samples were frozen in liquid nitrogen, pulverized, and thoroughly homogenized in QIAzol lysis reagent (QIAGEN, Dusseldorf, Germany). Total RNA was isolated using TRIzol reagent (Invitrogen, Carlsbad, CA, USA). The concentration of the RNA was determined using a spectrophotometer. Complementary DNA was synthesized from 0.5 *μ*g of total RNA using the SuperScript III First-Strand Synthesis System (Invitrogen). The mRNA expression levels of the osteoblastic markers RUNX2, OPN, and OSX and of the adipogenesis-specific marker PPAR*γ*2 were quantified by real-time RT-PCR using the SYBR Premix Ex Taq Kit (Takara Bio, Shiga, Japan) and a LightCycler 480 II instrument (Roche Diagnostics, Basel, Switzerland). The glyceraldehyde-3-phosphate dehydrogenase (GAPDH) housekeeping gene was used as reference for normalization of mRNA levels. All PCR reactions were performed in triplicate. Primers used for real-time RT-PCR are listed in Table 1.

### 2.7. Histological Analysis

Isolated tibias were fixed in 10% neutral-buffered formalin, transferred to 70% ethanol, and decalcified in 9% formic acid. After tissue processing, specimens were embedded in paraffin. Sections (5 *μ*m) were cut sagittally along the tibial shaft axis and collected on glass slides before being deparaffinized and subjected to hematoxylin and eosin (H&E) staining using standard protocols. After mounting with coverslips, specimens were viewed and analyzed under a light microscope.

### 2.8. Statistical Analysis

Data are expressed as means ± SD. Significant differences were analyzed by Student's *t*-test or one-way ANOVA followed by* post hoc* Newman-Keuls multiple comparison test using GraphPad Prism 6.0 software. *P* values less than 0.05 were considered significant.

## 3. Results

### 3.1. Determination of BMD in SD Rats with Osteoporosis

The BMDs of ovariectomized rats were determined* in vivo*. There was no significant difference in BMD among the experimental groups before treatment. Additionally, there was no difference in sham group BMDs before and after treatment. There was, however, a significant difference in BMD between sham and ovariectomized rats (*P* < 0.05). Moreover, compared to preoperative BMD values, ovariectomized groups exhibited reduced BMDs 12 weeks after surgery (*P* < 0.01), indicating that we were successful in inducing an osteoporosis model (Figure 1(b)). After surgery, no mortality or femoral fractures were observed throughout the study. All rats were able to walk normally 20 h after surgery.

### 3.2. Characterization of Rat BMSCs and the Effect of PRP on Cells

**Figure 2:**
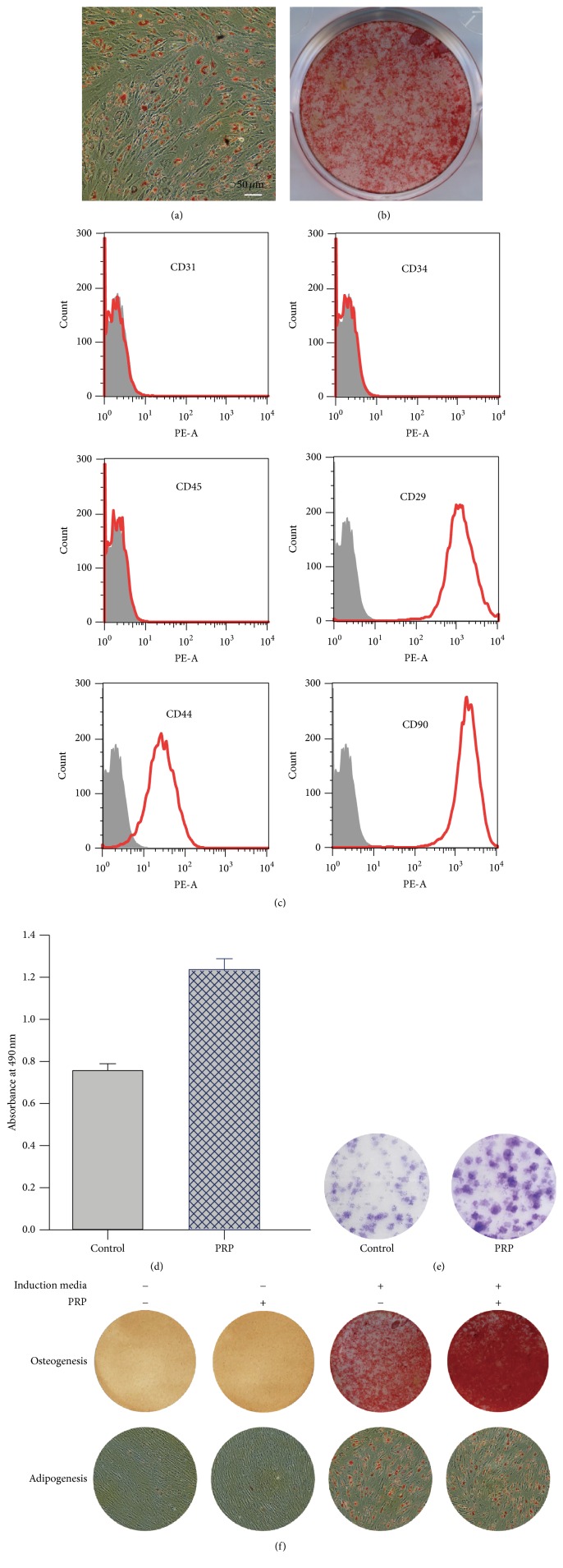
Differentiation of rat bone marrow mesenchymal stem cells (BMSCs) into adipocytes and osteoblasts. Isolated rat BMSCs were grown in either adipogenic or osteogenic induction media, and their adipogenic and osteogenic differentiation abilities were assessed by oil red O staining (a) and alizarin red staining (b), respectively. (c) Phenotypic characterization of the rat BMSCs at passage 3. (d) Effect of PRP on the proliferation of rat BMSCs. Rat BMSCs were cultured in complete growth media with or without PRP for 48 h, and the proliferation of cells was measured by MTS assays. (e) The colony-forming efficiency of rat BMSCs is enhanced by PRP treatment. The cells were cultured in complete growth media with or without PRP for 2 weeks, and the colony-forming efficiency was determined by the colony formation assay. (f) Effects of PRP on the adipogenic (lower panel) and osteogenic (upper panel) differentiation potentials in rat BMSCs.

Pretreated marrow cells were plated into flasks and were cultured for approximately two weeks. Typical spindle-shaped cells appeared and began to grow and divide 72 h after seeding. Colonies appeared after 6–9 days and became confluent after 2-3 weeks (data not shown). Rat BMSCs isolated at passages 2 and 4 were analyzed for their capacity to differentiate following adipogenic and osteogenic induction. Lipid droplets were visualized by staining with oil red O (Figure 2(a)), and calcium deposition was determined by staining with alizarin red (Figure 2(b)). Based on analysis of surface marker expression using flow cytometry, cells were negative for CD31, CD34, and CD45 and positive for CD29, CD44, and CD90 (Figure 2(c)). Next, to examine the effect of PRP on the cell growth (proliferation and colony formation) and differentiation capacities* in vitro*, BMSCs were treated with or without PRP, and the MTS assays, colony-forming assays, and adipogenic/osteogenic differentiation assays were performed. We observed that the number of cells and colonies formed in PRP-containing media was more than that in control media (Figures 2(d) and 2(e)). Meanwhile, while PRP alone failed to induce osteogenesis as demonstrated by alizarin red staining, the addition of PRP to osteogenic medium dramatically increased the number of alizarin red-positive cells (Figure 2(f), upper). Furthermore, oil red O staining revealed that PRP was not able to trigger adipogenesis when used alone and had no noticeable effect on adipogenic differentiation when added to adipocyte-inducing media (Figure 2(f), lower).

### 3.3. *In Vivo* Micro-CT Analysis of New Bone Formation at Defect Sites

**Figure 3:**
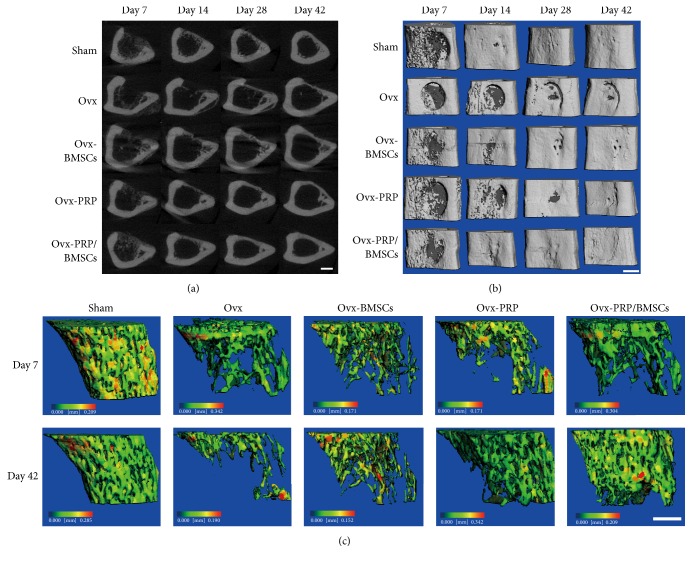
Temporal micro-CT analysis of bone healing. Representative 2D (a) and 3D (b) images were generated by micro-CT showing the bone healing process after drill-hole surgery. Scale bar, 1 mm. (c) Trabecular bone volumes of proximal tibial growth plates were assessed by micro-CT scan. Scale bar, 1 mm.

Bone healing processes in the areas surrounding the defects were monitored by* in vivo* micro-CT scan 7, 14, 28, and 42 d after injury. Midpoint coronal plane 2D (Figure 3(a)) and 3D (Figure 3(b)) images were acquired and reconstructed. Hematomas were visible in bone defect areas in sham, Ovx-BMSC, Ovx-PRP, and Ovx-PRP/BMSC rats 7 d after injury, while there were no obvious hematomas in the Ovx group. By day 14 after injury, bone defects in sham and Ovx-PRP/BMSC rats had mostly closed due to newly formed calluses. In contrast, only thin calluses had formed in Ovx-BMSC and Ovx-PRP rats, and there were no obvious calluses at all in Ovx rats. By day 28 after injury, bone defects in sham and Ovx-PRP/BMSC rats had been completely closed by calluses with high density and mineralization. While the bone defect areas in the other three groups were mostly covered by calluses, callus density was relatively low. By 42 days after injury, bone defects had completely healed in sham and Ovx-PRP/BMSC rats, and calluses had mostly mineralized according to the 2D coronal images. There was no discernable difference between the callus and the surrounding bone tissue. In Ovx-BMSC and Ovx-PRP rats, calluses had thickened and the degree of mineralization increased. In contrast, the bone defect had not completely closed in Ovx rats.

In order to identify changes to bone trabeculae besides those at the defect site, micro-CT scans were taken of the proximal tibial growth plate. There were no obvious changes to bone trabeculae in Ovx or Ovx-BMSC rats from day 7 to day 42 after surgery. However, the bone volumes of Ovx-PRP and Ovx-PRP/BMSC rats significantly increased over this time period (Figure 3(c)). The BV/TV, Tb.N, and Conn.D values in the proximal tibias were higher in the sham group than those in the other groups. In both Ovx-PRP and Ovx-PRP/BMSCs groups, the BV/TV, Tb.N, and Conn.D values of the defect areas increased significantly from day 7 to day 42, whereas the Tb.N and Conn.D values in Ovx rats decreased during the bone reproduction process (Table 2).

### 3.4. Quantitative Real-Time PCR Analysis of Gene Expression during Bone Healing

**Figure 4:**
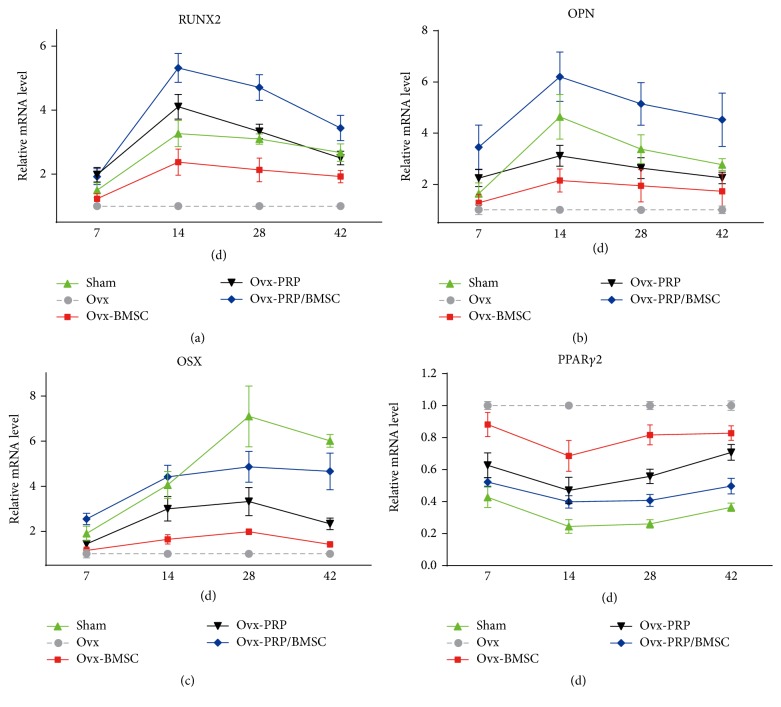
Temporal analysis of callus gene expression. Temporal changes in RUNX2, OPN, OSX, and PPAR*γ*2 mRNA expression levels in callus samples after drill-hole surgery were determined by quantitative real-time PCR. Expression levels were normalized to that of GAPDH, and levels were calculated as fold-change values relative to that of the Ovx group. Data are reported as means ± SD, *n* = 5.

The mRNA expression levels of RUNX2, OPN, OSX, and PPAR*γ*2 in the calluses of sham, Ovx-BMSC, Ovx-PRP, and Ovx-PRP/BMSCs rats were determined as fold-change values relative to those of the Ovx group. Expression levels of RUNX2 and OPN steadily increased from day 7, peaking at day 14 before decreasing from day 14 to day 42 (Figures 4(a) and 4(b)). While expression levels of OSX steadily increased from day 7 to day 28 and decreased thereafter in all groups, levels were significantly lower in Ovx-BMSC rats than those in Ovx-PRP/BMSC rats on days 14 and 28 (Figure 4(c)). Expression levels of PPAR*γ*2 were downregulated in sham, Ovx-BMSC, Ovx-PRP, and Ovx-PRP/BMSC rats compared to those in Ovx rats, with Ovx-PRP/BMSC rats exhibiting even lower expression levels than those of Ovx-BMSC and Ovx-PRP rats (Figure 4(d)).

### 3.5. Histological Analysis of Bone Healing

**Figure 5:**
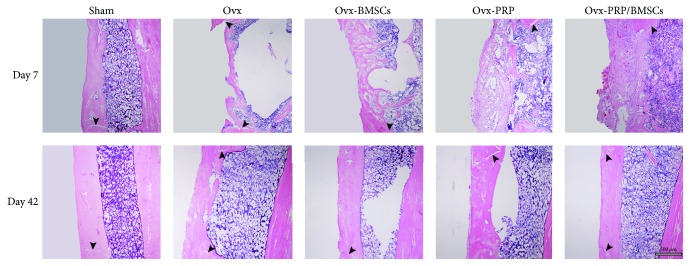
Histological analysis of bone healing progression. Representative photomicrographs of callus sections from all groups demonstrate bone healing after drill-hole surgery. Callus sections extracted on the indicated day after surgery were stained with hematoxylin and eosin. Arrows indicate cortical gaps.

By 7 d after induction of bone defects, there was a predominance of soft tissue composed of a mixture of hematoma and granulation tissue in Ovx-BMSCs and Ovx-PRP rats. The bone defect area was filled with an irregular fibrous callus in the sham group. Newly woven bone was identified across the intramedullary region and cortical gap in Ovx-PRP/BMSCs rats. However, the density and volume of these bone structures were lower in the Ovx-PRP/BMSCs group than those in the sham group. By day 42, bone defect sites were completely filled with calluses in all groups. The thickness of each callus was equal to that of the adjacent cortical bone and lamellar bone had fully formed parallel to the backbone in sham and Ovx-PRP/BMSCs rats, whereas woven bone remained in Ovx, Ovx-PRP, and Ovx-BMSCs rats. The cortical gap could be identified in all groups (Figure 5).

## 4. Discussion

Osteoporosis is a systemic skeletal disease characterized by a decrease in bone mass as well as a deterioration of bone architecture resulting in an increased risk of fractures [18, 19]. Reduced estrogen levels in postmenopausal women along with declines in BMSC number and osteogenic differentiation capacity are important contributors to age-related bone loss [20]. PRP has been widely used in various clinical procedures due to its high concentration of growth factors and bioactive proteins that influence the healing of bone injuries [7, 21]. Although not yet conclusive, it appears that most research supports a positive role for PRP in bone regeneration* in vivo* [22, 23]. However, virtually all the previous studies were done in normal animals, and it is not clear whether the impact of PRP would be compromised by osteoporosis. In the present study, by using a well-established animal model of osteoporotic bone defects, we demonstrated that treatment with PRP combined with BMSCs can be used to enhance bone healing. To our knowledge, this is the first study of its kind to examine the efficacy of a combination of PRP and BMSCs treatment in an osteoporotic bone defect animal model.

In order to explore the potential mechanisms by which PRP and BMSCs accelerated bone regeneration, we analyzed the expression levels of osteogenesis-related genes RUNX2, OPN, and OSX and of the major adipogenesis regulator PPAR*γ*2 during bone healing. We found that RUNX2, OPN, and OSX were generally upregulated following PRP and/or BMSC treatment. Notably, the highest expression levels of these genes were observed in the Ovx-PRP/BMSCs group, indicating that the positive effect of BMSCs on bone formation might be enhanced in the presence of PRP. Since PRP remarkably promoted the growth and osteogenic differentiation capacities of BMSCs* in vitro*, it is thus possible that PRP may accelerate bone repair by upregulating the proliferation and osteogenesis of BMSCs. In contrast, PPAR*γ*2 expression was suppressed, particularly in Ovx-PRP/BMSCs rats. These results suggest that PRP is involved in mediating the balance between osteogenesis and adipogenesis* in vivo*, which somehow may also contribute to the positive effects of PRP on bone formation in osteoporotic rats.

Micro-CT has been shown to be a useful tool for precisely measuring changes in bone stereology, volume, and microarchitecture [24]. During the late phase of bone healing, the newly formed bone in the defect area was fully remodeled into a cortical-like structure in sham and Ovx-PRP/BMSCs rats, whereas remodeling was incomplete in Ovx-PRP, Ovx-BMSCs, and Ovx rats, as observed in both 2D and 3D images. The lamellar bone of defect sites in Ovx-PRP and Ovx-BMSCs rats was relatively thin, and the degree of healing was comparable to that of Ovx rats. These results indicate that the remodeling capacity from woven bone to lamellar bone can be affected by various treatments. This finding is consistent with that of a previous study involving an estrogen-deficient mouse model treated with murine embryonic stem cell-derived osteoblasts [25]. Furthermore, 3D reconstructions of proximal tibial growth plates revealed that bone volume was significantly increased in Ovx-PRP/BMSCs and Ovx-PRP rats compared with that in Ovx rats. These data reinforce the notion that PRP may be useful in enhancing bone volume and treating pathological conditions of bone loss.

## 5. Conclusions

Although both micro-CT and histological analysis confirmed that PRP and BMSC treatment is beneficial for bone healing, these analyses were not able to determine to what extent and for how long the exogenous cells act on the bone injury site. Therefore, further experiments should be conducted using alternative* in vivo* cell tracking techniques, such as labeling with superparamagnetic iron oxide or cell tracking dyes. These analyses would facilitate the monitoring and identification of introduced exogenous cells.

In conclusion, our results suggest that PRP and BMSCs promote new bone formation. Better results were obtained by combining the two into a single treatment for osteoporotic bone defects. PRP can promote the proliferation and osteogenic differentiation of BMSCs, which may play an important role in the initiation of bone repair. Our findings may provide insight for the development of a novel therapeutic strategy in osteoporotic bone healing.

## Figures and Tables

**Table 1:** Oligonucleotide primer sequences for quantitative real-time PCR analysis of gene expression.

Target gene	Sequences (5′-3′)	Product size (bp)	GenBank accession number
GAPDH	Forward, TGCCACTCAGAAGACTGTGG	129	AF106860
	Reverse, TTCAGCTCTGGGATGACCTT		
RUNX2	Forward, CCACCACTCACTACCACACG	119	NM_001278483
	Reverse, GGACGCTGACGAAGTACCAT		
OPN	Forward, GATCGATAGTGCCGAGAAGC	111	M99252
	Reverse, TGAAACTCGTGGCTCTGATG		
OSX	Forward, CCAATGACTACCCACCCTTTC	109	AY177399
	Reverse, ACACTAGGCAGGCAGTCAGAA		
PPAR*γ*2	Forward, GGTTGACACAGAGATGCCATT	123	NM_001145367
	Reverse, CTGGAGAAATCAACCGTGGTA		

**Table 2:** Micro-CT analysis of bone microstructure changes in proximal tibial growth plates after surgery ($\overset{-}{x}\pm s$, *n* = 5).

Group	Time	Tb.N (1/mm)	Tb.Sp (mm)	BV/TV (%)	Conn.D
Sham	Day 7	4.867 ± 0.187	0.166 ± 0.012	0.445 ± 0.027	91.545 ± 6.281
	Day 42	4.759 ± 0.123	0.177 ± 0.003	0.383 ± 0.029	91.598 ± 6.743
Ovx	Day 7	1.518 ± 0.114	0.700 ± 0.051	0.128 ± 0.060	12.434 ± 3.144
	Day 42	0.823 ± 0.203^*∗*^	1.277 ± 0.290	0.048 ± 0.014	3.425 ± 0.478^*∗*^
Ovx-BMSC	Day 7	1.321 ± 0.108	0.655 ± 0.202	0.115 ± 0.043	10.440 ± 3.321
	Day 42	1.438 ± 0.288	0.295 ± 0.082	0.154 ± 0.028	15.997 ± 3.361
Ovx-PRP	Day 7	1.567 ± 0.079	0.678 ± 0.034	0.118 ± 0.055	13.377 ± 5.644
	Day 42	3.838 ± 0.471^*∗*^	0.266 ± 0.041^*∗∗*^	0.194 ± 0.055^*∗*^	46.836 ± 9.086^*∗*^
Ovx-PRP/BMSC	Day 7	1.658 ± 0.501	0.663 ± 0.211	0.131 ± 0.068	15.870 ± 5.861
	Day 42	4.242 ± 0.057^*∗∗*^	0.219 ± 0.005^*∗*^	0.282 ± 0.038^*∗*^	76.024 ± 6.563^*∗∗*^

^*∗*^ *P* < 0.05 and ^*∗∗*^ *P* < 0.01 versus day 3 values.
